# The *Mycobacterium tuberculosis* specific antigen MPT64 in BALF has potential diagnostic value in the diagnosis of pulmonary tuberculosis

**DOI:** 10.3389/fmed.2026.1726316

**Published:** 2026-02-11

**Authors:** Jie Sun, Weiwei Lin, Shiyu Fang, Jinxiong Jiang, Fengjun Liu

**Affiliations:** 1 Department of Infectious Diseases of Affiliated Hospital of North Sichuan Medical College, Nanchong, Sichuan, China; 2 North Sichuan Medical College, Nanchong, Sichuan, China

**Keywords:** BALF, enzyme-linked immunosorbent assay, MPT64, tuberculosis, tuberculosis antigen

## Abstract

**Background:**

Recent research on the use of the MPT64 antigen of *Mycobacterium tuberculosis (MTB)* in tuberculosis diagnosis has intensified. However, its detection in bronchoalveolar lavage fluid (BALF) has not been previously documented. This study aims to fill that gap.

**Methods:**

We included a total of 176 patients, divided into a pulmonary tuberculosis (PTB) group of 104 cases and a non-tuberculosis (Non-TB) group of 72 cases as the control group. The PTB group includes 59 with bacteriologically confirmed PTB (BC-PTB) and 45 with clinically diagnosed PTB with negative pathogens (CD-PTB). The concentrations of MPT64 antigens were detected by enzyme-linked immunosorbent assay (ELISA). Optimal cut-off values were determined by receiver operating characteristic (ROC) curves to evaluate antigen diagnostic capability for active PTB, compared with acid-fast bacilli (AFB) and Xpert MTB/RIF.

**Results:**

Xpert MTB/RIF Ct values and MPT64 concentration show significant negative correlation (*R* = -0.719, *P* < 0.0001), AFB and MPT64 concentration show significant positive correlation (R = 0.777, *P* < 0.0001). MPT64 showed a sensitivity of 59.62% (95% CI: 50.01–68.54%) in 104 cases, significantly higher than AFB (26.92%, *P* < 0.001) and slightly higher than Xpert MTB/RIF (56.73%, *P* = 0.673). Its specificity was 88.89% (95% CI: 79.58–94.26%), lower than both AFB and Xpert MTB/RIF (100%, *P* = 0.006). Sensitivities for BC-PTB and CD-PTB were 64.41% (95% CI: 51.66–75.40%) and 53.33% (95% CI: 39.08–67.06%), respectively, *P* = 0.314.

**Conclusion:**

The use of ELISA to detect the MPT64 in BALF may serve as an important supplementary diagnostic method for pulmonary tuberculosis, particularly for bacterial-negative pulmonary tuberculosis.

## Introduction

Tuberculosis is an infectious disease caused by *Mycobacterium tuberculosis (MTB)* that poses a serious threat to human health. The World Health Organization’s Global Tuberculosis Report 2024 (1) indicates that in 2023 there were 10.8 million new tuberculosis cases worldwide, with 1.25 million deaths, making tuberculosis the world’s deadliest infectious disease. Early diagnosis of active tuberculosis is critical for effective treatment and prevention of disease transmission. Pathogenic testing remains the cornerstone for the diagnosis of pulmonary tuberculosis. Currently, clinical methods include acid-fast bacilli (AFB) staining, detection of *MTB* nucleic acids, and *MTB* culture. Although AFB staining is convenient, its positive rate is low and it cannot reliably differentiate between *MTB* and *non-tuberculous mycobacteria.* Xpert MTB/RIF, which detects *MTB* nucleic acid with higher sensitivity (2) and can assess rifampicin resistance, requires sophisticated equipment and skilled operators, limiting its use in underdeveloped areas. Moreover, the detection of dead *MTB* DNA fragments poses challenges in evaluating bacterial activity, TB lesion activity, and treatment follow-up (3, 4). *MTB* culture, while the gold standard, is time-consuming due to the slow growth of the organism and requires biosafety Level II laboratories, making it impractical in resource-limited settings (5–7). Therefore, a new, rapid, and simple complementary method for tuberculosis diagnosis is warranted.

Recent research on *MTB*-specific antigens has revealed that secreted proteins provide direct evidence of active *MTB* infection and may help distinguish current from past infection (8). MPT64 is a 24-kDa protein secreted by *MTB* during its active growth phase, this antigen is absent in *non-tuberculous mycobacteria* and the BCG strain, which lacks the RD2 region (9, 10). Has been extensively used to identify culture-positive *MTB* through immunohistochemistry and immunocytochemistry (11). Owing to its excellent specificity, detection of the MPT64 antigen aids in identifying viable *MTB*. Literature indicates that MPT64 antigen detection has significant diagnostic value in various extrapulmonary specimens, as confirmed by cohort-based diagnostic accuracy studies (12–14).

Although sputum examination is a commonly used method for tuberculosis diagnosis, its limitations—such as atypical clinical symptoms in some patients, insufficient sputum samples, and low bacterial loads—can delay diagnosis and treatment, especially in bacterial-negative cases (15, 16). In contrast, BALF directly targets lesions in the lower respiratory tract, thereby significantly improving the detection rate of pulmonary pathogens and garnering increased attention in clinical practice (17). Several studies have demonstrated that the diagnostic sensitivity of BALF is superior to that of sputum for AFB staining, PCR, and *MTB* culture (17, 18). For liquid samples, ELISA is considered the preferred method because of its ease of operation, rapidity, and minimal equipment and operator requirements. Therefore, this study aimed to use ELISA to detect the *MTB* antigen MPT64 in BALF and evaluate its potential value in diagnosing pulmonary tuberculosis. To date, no study has reported on the use of BALF to detect the *MTB*-specific antigen MPT64, this study is the first to detect, thereby addressing a gap in the existing literature.

## Materials and methods

### Study design and participants

This retrospective study enrolled 176 patients who underwent fiber bronchoscopy and alveolar lavage at the Affiliated Hospital of North Sichuan Medical College from May 2021 to July 2023. Indications for fiber bronchoscopy examination (meeting one or more criteria): (1) Patients with suspected symptoms of tuberculosis, such as fever, night sweats, cough, sputum production, and weight loss, at all; (2) Patients with lesions on lung X-rays or CT scans.

Among the collected cases, 104 patients diagnosed with pulmonary tuberculosis were selected as the study subjects (pulmonary tuberculosis group, PTB). The diagnostic criteria for tuberculosis were based on the Health Industry Standard of the People’s Republic of China WS288–2017 (19). This includes, (1) 59 cases of bacteriologically confirmed pulmonary TB (BC-PTB): Two specimens of BALF were AFB stained or Xpert MTB/RIF positive; (2) 45 were clinically diagnosed pulmonary tuberculosis (CD-PTB): The Xpert MTB/RIF and AFB staining of specimens are both negative, and other lung diseases are excluded after differentiation, while meeting one or more of the following criteria: Patients with lesions on lung X-rays or CT scans and suspected clinical manifestations of pulmonary tuberculosis; The lung X-ray or CT shows lesions and the interferon-γ release assay (IGRA) result is positive; Fibrobronchial examination reveals lesions in the trachea or bronchi; Antituberculosis therapy is effect.

As the control group, 72 patients had no history of tuberculosis, and the IGRA result was negative (non-tuberculosis group, Non-TB). The pulmonary lesions were excluded from tuberculosis, and no tuberculosis was diagnosed after follow-up.

### Methods

#### Collection and preservation of BALF

After obtaining informed consent, BALF collection strictly adhered to technical. guidelines provided by the European Respiratory Society (20, 21) and standard operating procedures. Alveolar lavage involved three lavages using sterile saline, each with a volume of 20–60 mL, totaling 100–300 mL. Fluid recovery was conducted at a negative pressure of -3.3 to -13.3 kPa after each lavage, achieving a recovery rate of 40–70%. The collected fluid was immediately centrifuged at 500 g for 15 min to pellet cellular debris. The cell-free supernatant was then carefully aliquoted into sterile frozen tubes pretreated with silicone oil, and immediately stored at -20°C. To prevent sample degradation, each sample underwent a single freeze-thaw cycle, with thawing performed overnight at 4°C before use.

#### Determination of optimal sample dilution

Determine the optimal sample dilution using chessboard titration method. Select three samples with the highest content of acid fast bacilli (smear results from +++ to +++++), and pre-test four dilution ratios of 1:1, 1:5, 1:10, and 1:20. Determine the optimal dilution by calculating the absorbance ratio (P/N value) between the positive sample and the negative control. He experimental results showed that the undiluted sample had the highest P/N value, so subsequent tests were conducted using the original liquid sample.

#### ELISA detection of MPT64

The human *Mycobacterium tuberculosis* ELISA Kit (Camilo Biological Company, Nanjing, China) was used according to the manufacturer’s instructions. The thawed BALF was centrifuged at 4°C for 10 min, and the supernatant was used for testing. After equilibrating the kit to room temperature, the freeze-dried human MPT64 standard was reconstituted with 1.0 mL of standard dilution solution to produce a stock solution. This stock solution was serially diluted to obtain concentrations of 100, 50, 25, 12.5, and 6.3%, followed by further dilutions to 3.1, 1.5, and 0%. Standard wells and sample wells were then loaded (100 μL per well), and the plate was sealed and incubated for 90 min at 37°C. After washing twice, 100 μL of biotinylated human MPT64 antibody working solution was added, the plate was resealed and incubated at 37°C for 60 min. The plate was washed three times, and 100 μL of enzyme conjugate working solution was added to all wells (except blank wells). The plate was resealed and incubated at 37°C in the dark for 30 min. Following five washes, 100 μL of TBM color development working solution was added; incubation at 37°C continued until a clear color gradient was observed in the standard wells, after which 100 μL of stop solution was added immediately with mixing. The optical density (OD) values were measured using a Perlong microplate reader (Beijing). The average OD value of duplicate wells was used to construct a standard curve, ensuring that the coefficient of variation (CV) between wells was less than 10%. The OD value of the blank wells was subtracted from each standard well’s OD value.

#### Conventional laboratory examinations

The collected bronchoalveolar lavage fluid was routinely sent to the hospital laboratory for AFB and Xpert MTB/RIF testing, and the results were reviewed by two experienced technicians. The grading of AFB smear positivity followed the standards provided by the Health Industry Standard of the People’s Republic of China WS288–2017 (19), recorded as negative, (+), (++), and (+++). The Xpert MTB/RIF results were used to detect *MTB* and rifampicin resistance, with the minimum Ct value (threshold cycle number) of all detected probes recorded for each Xpert MTB/RIF test. 15 ≤ Ct ≤ 18.9 indicates a high detection of *MTB*,19 ≤ Ct ≤ 24.9 indicates a moderate detection of *MTB*,25 ≤ Ct ≤ 28.9 indicates a low detection of *MTB*, and 29 ≤ Ct ≤ 32 indicates a very low detection of *MTB*, Ct > 32 indicators a negative.

### Statistical analysis

The standard curve was constructed and analyzed using ELISACalc. SPSS25 software was employed for statistical analysis. The relationship between the OD values and PTB status was evaluated using receiver operating characteristic (ROC) curve analysis. For tuberculosis diagnosis, the optimal ELISA cutoff value was determined by maximizing the Youden index. Spearman rank correlation coefficient to evaluate the correlation between two sets of non-parametric data. Categorical variables were compared using the Pearson chi-square test or Fisher’s exact test, with a significance threshold set at *P* < 0.05.

## Results

### Clinical data

The basic characteristics of the patients are summarized in Table 1. Ages ranged from 13 to 85 years. Males constituted 75.3% (123/176) of the sample, and cough was the most common symptom, observed in 69.3% (122/176) of cases. Among PTB patients, diabetes was the most common comorbidity, present in 11.5% (12/104) of cases.

**TABLE 1:** Basic characteristics of participants (*n* = 176).

	PTB (*n* = 104)	Non-TB (*n* = 72)
Age (median)	44 (13–78)	58.5 (16–85)
**Gender**		
Male (%)	75 (72.1)	48 (66.7)
Female (%)	29 (27.9)	24 (33.3)
Prior history of tuberculosis (%)	1 (1.0)	0
**Symptom**		
Cough (%)	68 (65.3)	54 (75)
Fever (%)	14 (13.4)	23 (31.9)
Marasmus (%)	19 (18.2)	11 (15.2)
Hot flash and night sweats (%)	27 (25.9)	5 (6.9)
Asymptomatic (%)	18 (17.3)	12 (16.6)
**Major comorbidities**		
Diabetes mellitus (%)	12 (11.5)	9 (12.5)
Hypertension (%)	2 (1.9)	9 (12.5)
HIV (%)	0	4 (5.6)
Lung tumor (%)	3 (2.9)	8 (11.1)

PTB, pulmonary tuberculosis group; Non-TB, non-tuberculosis group.

### Correlation analysis of MPT64 concentration with Xpert MTB/RIF and AFB

**FIGURE 1:**
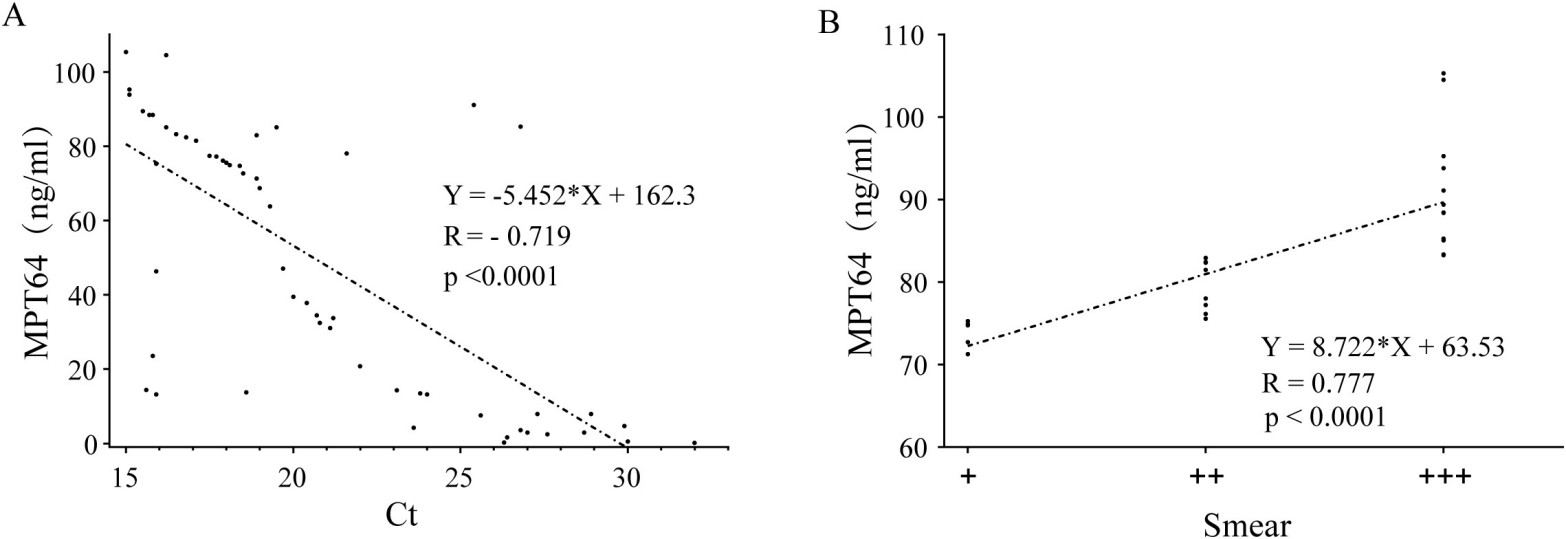
Correlation analysis of MPT64 concentration with Xpert MTB/RIF and AFB. **(A)** Correlation analysis of Xpert MTB/RIF Ct values and MPT64 concentration in the BC-PTB group. **(B)** Linear regression analysis between AFB smear grade and MPT64 concentration in the BC-PTB group.

In the BC-PTB group, we observed a strong, statistically significant negative correlation between Xpert MTB/RIF Ct values and MPT64 concentration (*R* = -0.719, *P* < 0.0001) (Figure 1A), this indicates that lower Ct values are strongly associated with higher levels of the MPT64 antigen.

The linear regression reveals a strong, positive, and statistically highly significant correlation between the AFB smear grade and the MPT64 concentration (*R* = 0.777, *P* < 0.0001) (Figure 1B). This indicates that the higher the AFB smear grade, the higher the concentration of MPT64.

### BALF MPT64 concentrations

**FIGURE 2:**
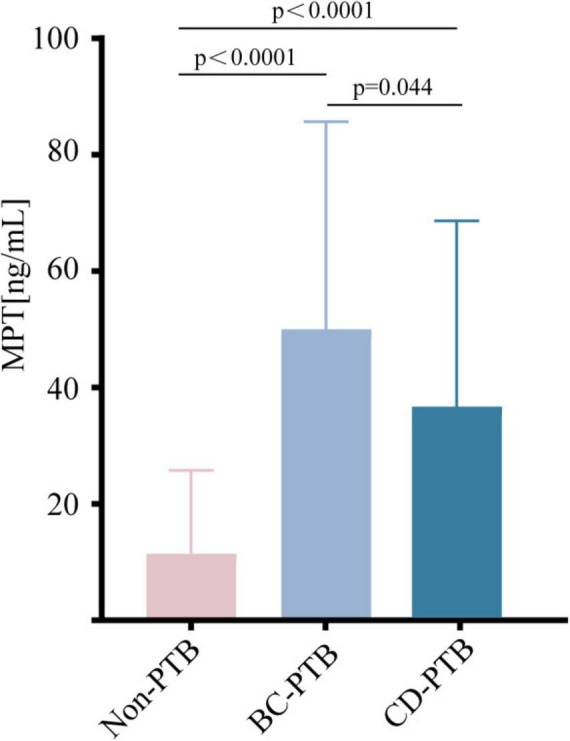
MPT64 concentration distribution diagram of non-TB group and PTB group. The number of subjects in each group (Non-TB: *n* = 72; BC-PTB: *n* = 59; CD-PTB: *n* = 45), indicates that between-group comparisons were performed using the Mann-Whitney U test, and specifies that error bars represent the interquartile range (IQR). Non-TB represents the non-tuberculosis group; PTB represents the pulmonary tuberculosis group; BC-PTB represents bacteriologically confirmed pulmonary tuberculosis; CD-PTB represents clinically diagnosed pulmonary tuberculosis.

Based on the ELISA standard curve, the protein concentration in each sample was calculated. The analysis demonstrated that the concentration of MPT64 in the non-TB group was significantly lower than in the BC-PTB and CD-PTB groups (*P* < 0.0001). However, MPT64 levels were higher in BC-PTB than CD-PTB groups (*P* = 0.044) (Figure 2).

**FIGURE 3:**
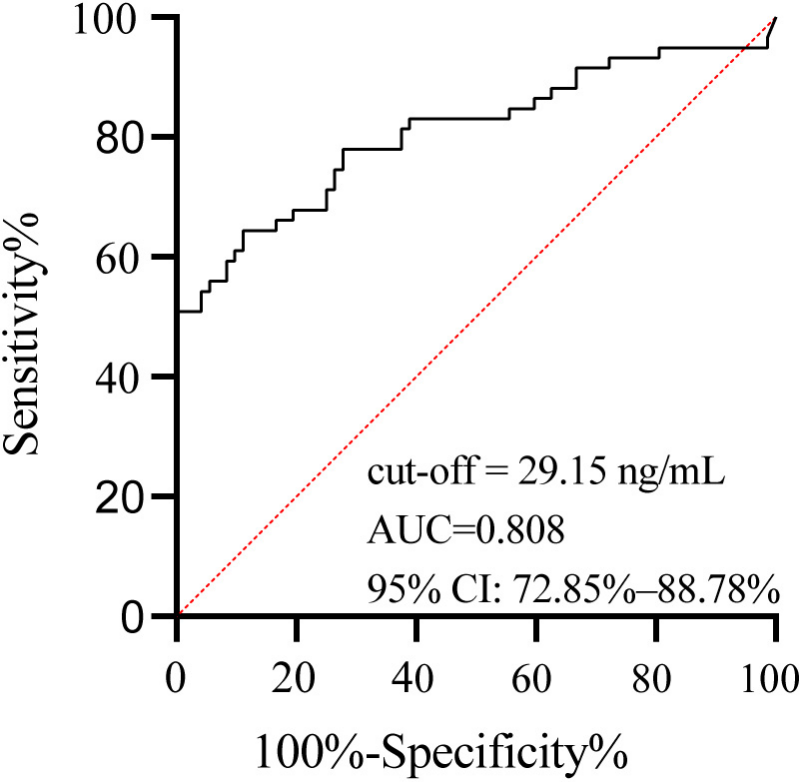
ROC curve of MPT64 concentration in the BC-PTB group. The area under the curve (AUC) for the MPT64 antigen concentration was 0.808 (95% CI: 72.85–88.78%). The optimal cutoff, determined by the maximum Youden index, was 0.533, corresponding to an antigen concentration of 29.15 ng/mL.

The ROC curve was constructed for the PTB group (Figure 3). The area under the curve (AUC) for the MPT64 antigen concentration was 0.808 (95% CI: 72.85–88.78%). The optimal cutoff, determined by the maximum Youden index, was 0.533, corresponding to an antigen concentration of 29.15 ng/mL. This cutoff was then used to calculate the sensitivity and specificity of MPT64 detection in all tuberculosis groups (Table 2).

**TABLE 2:** Diagnostic value of MPT64, AFB, and Xpert MTB/RIF for tuberculosis (*n* = 176).

		PTB (*n* = 104)	Non-TB (*n* = 72)	Sensitivity (%)	Specificity (%)	PPV (%)	NPV (%)
AFB	+	28	0	26.92 (18.45–35.39)	100 (93.75–100)	100 (87.93–100)	48.65 (40.69–56.60)
	-	76	72				
Xpert MTB/RIF	+	59	0	56.73 (47.45–66.20)	100 (93.75–100)	100 (93.88–100)	61.54 (52.72–70.35)
	-	45	72				
MPT64	+	62	8	59.62 (50.20–69.13)	88.89 (79.32–96.14)	88.57 (79.04–94.10)	60.38 (51.07–69.69)
	-	42	64				

AFB, acid-fast staining; Xpert MTB/RIF, MTB complex rifampicin resistance gene detection; MPT64, tuberculosis specific antigen MPT64; PTB, pulmonary tuberculosis group; Non-TB, non-tuberculosis group; PPV, positive predictive value; NPV, negative predictive value.

### Comparison of MPT64 with AFB staining and Xpert MTB/RIF

**FIGURE 4:**
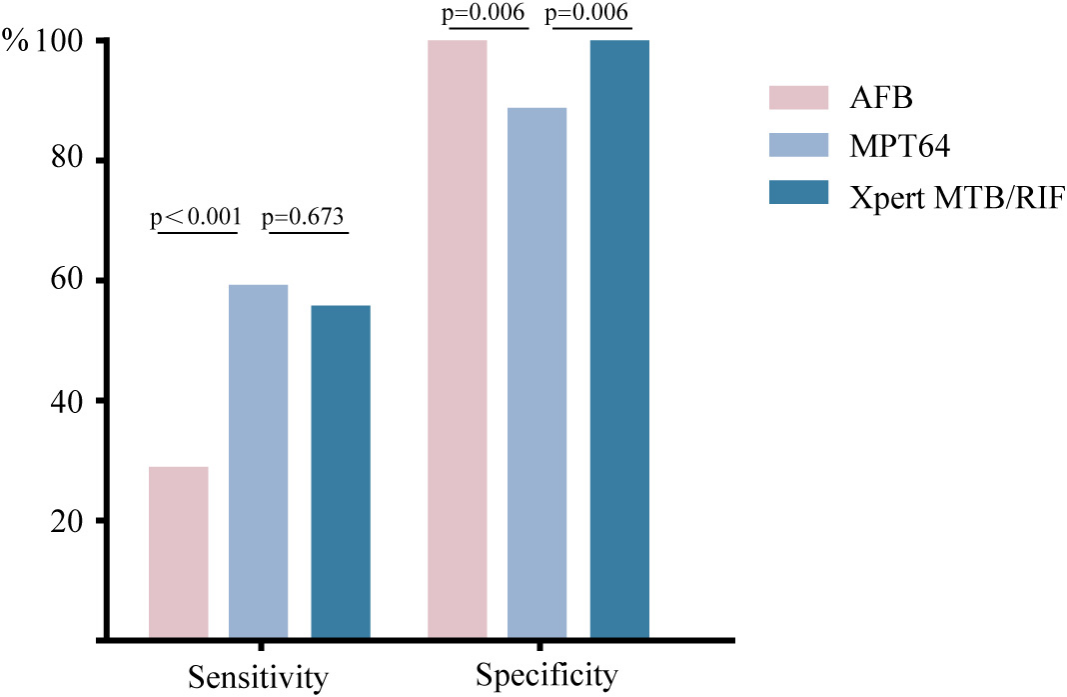
Sensitivity and specificity of MPT64, AFB, and Xpert MTB/RIF. The sensitivity and specificity values were derived using the 29.15 ng/mL cut-off for MPT64.

As shown in Table 2, Compared with AFB, the sensitivity of MPT64 detection increased by 32.7% (*P* < 0.001), which was statistically significant. In comparison with Xpert MTB/RIF, the sensitivity increased by 2.89% (*P* = 0.673), which was not statistically significant. In terms of specificity, MPT64 detection was 11.11% lower than that of both AFB and Xpert MTB/RIF, and this difference was statistically significant (Figure 4).

### Diagnostic value of MPT64 in the BC-PTB and CD-PTB groups

Among the 59 BC-PTB cases, 38 tested positive for the MPT64 antigen, yielding a sensitivity of 64.41% (95% CI: 51.66–75.40%). In the 45 CD-PTB cases, 24 tested positive, resulting in a sensitivity of 53.33% (95% CI: 39.08–67.06%). The difference in sensitivity between these two groups was not statistically significant (*P* = 0.314) (Table 3).

**TABLE 3:** Sensitivity of MPT64 in the BC-PTB and CD-PTB groups.

	MPT64 +	MPT64 -	Sensitivity (%)	*P*
BC-PTB (*n* = 59)	38	21	64.41 (51.66–75.40%)	0.314
CD-PTB (*n* = 45)	24	21	53.33 (39.08–67.06%)	

BC-PTB, bacteriologically confirmed PTB; CD-PTB, clinically diagnosed PTB.

## Discussion

To further elucidate the relationship between mycobacterial load and MPT64 antigen levels, we employed two clinically relevant surrogate measures: semi-quantitative AFB smear grading and Xpert MTB/RIF Ct values. Our analysis revealed that lower Ct values and higher smear grades were associated with a corresponding rise in MPT64 concentration. These strong correlations indicate that MPT64 levels directly reflect the mycobacterial burden in the lungs, validating its role as a biologically relevant marker and supporting its potential as a diagnostic tool for tuberculosis.

The results of this study demonstrate that the sensitivity of MPT64 detection in the PTB group (59.62%) is significantly higher than that of AFB staining and marginally higher than that of Xpert MTB/RIF. Although the specificity of MPT64 (88.89%) did not reach the 100% observed with AFB staining and Xpert MTB/RIF, its ability to reflect *MTB* activity via secretory properties offers unique advantages. These findings suggest that MPT64 detection could serve as an effective supplementary tool for the etiological diagnosis of tuberculosis. Previous studies have reported sensitivities of 88.00–100% and specificities of 96.4–100% for MPT64 detection using ultrasensitive ELISA on MTB cultures (22–24). The lower sensitivity and specificity observed in this study may be attributed to the lower bacterial load in BALF compared to culture media and the presence of proteinaceous substances in BALF that may lead to false-positive results. Furthermore, differences in detection methods might also account for these discrepancies, suggesting that enhancements in detection techniques could further improve sensitivity.

Comparing the sensitivity of MPT64 in BC-PTB and CD-PTB groups, there was no statistically significant difference (*P* = 0.314), suggesting that MPT64 antigen detection is less dependent on bacterial load than microbial methods, it has potential diagnostic value in the diagnosis of bacterial negative pulmonary tuberculosis. The complex interaction between *MTB* and the host, particularly regarding the mechanisms of bacterial protein secretion, remains only partially understood. Current research indicates that *MTB* employs a specialized secretory system to transfer specific proteins (24–26). These secreted proteins are usually smaller than the bacteria itself, and some of them can spread through the gaps or cells in the lung tissue to reach a distance from the bacterial body; the other part of the antigen may bind to the host’s immune cells and be transported to other parts (27–29). Previous studies have shown that *MTB* antigens are more diffusely distributed in lung tissues than the bacteria themselves (30, 31), suggesting that antigen detection may be more sensitive than direct bacterial detection. This supports the potential of using MPT64 detection in BALF as an auxiliary diagnostic method, particularly for bacterial-negative pulmonary tuberculosis. Given its simplicity, rapidity, cost-effectiveness, and minimal infrastructure requirements, the ELISA-based detection of MPT64 could be implemented widely, including in primary care settings.

There are some limitations to this article, including the lack of a healthy control group, as bronchoscopy is an invasive procedure and routine examinations are not recommended for healthy individuals; In addition, this study is a single center study with a relatively small sample size. In the future, a multi-center study can be conducted to increase the sample size to verify the critical value and standardize the protocol.

Diabetes is a common complication, which may have a potential impact on our core findings. Because the sample size is too small, no additional grouping of diabetes patients is conducted in this study. We will collect a larger sample size in the future.

## Conclusion

ELISA-based detection of the *MTB*-specific antigen MPT64 in BALF shows promise as an important supplementary diagnostic method for pulmonary tuberculosis, particularly in cases of bacterial-negative tuberculosis.

## Data Availability

The raw data supporting the conclusions of this article will be made available by the authors, without undue reservation.
